# Tools for generating and analyzing glycan microarray data

**DOI:** 10.3762/bjoc.16.187

**Published:** 2020-09-10

**Authors:** Akul Y Mehta, Jamie Heimburg-Molinaro, Richard D Cummings

**Affiliations:** 1 Department of Surgery, Beth Israel Deaconess Medical Center, National Center for Functional Glycomics, Harvard Medical School, Boston, MA, 02215, USA

**Keywords:** data analysis, glycan binding, glycan microarray, glycomics, informatics

## Abstract

Glycans are one of the major biological polymers found in the mammalian body. They play a vital role in a number of physiologic and pathologic conditions. Glycan microarrays allow a plethora of information to be obtained on protein–glycan binding interactions. In this review, we describe the intricacies of the generation of glycan microarray data and the experimental methods for studying binding. We highlight the importance of this knowledge before moving on to the data analysis. We then highlight a number of tools for the analysis of glycan microarray data such as data repositories, data visualization and manual analysis tools, automated analysis tools and structural informatics tools.

## Introduction

Glycans represent a major type of biomolecule in all living things, along with DNA, RNA, lipids and proteins [1]. In mammals, glycans commonly occur as post-translational modifications of proteins (glycoproteins), but they are also linked to lipids (glycolipids) and occur as free molecules. Such glycomolecules have vital roles in a wide range of physiological functions and also participate in many pathologic conditions [2]. Some classic examples of important glycans include the blood group antigens (A, B, O), which are glycan structures found on blood cells and tissues that play a critical role in determining transfusion compatibility during blood and organ donation [3], sialyl-Lewis^A^ antigen, known more commonly as CA19-9, which is a known tumor marker for pancreatic cancer, and could possibly promote cancer [4], and the *O*-glycan of PSGL-1 which is recognized by P- and L-selectin, which is critical for leukocyte recruitment [5–6]. Other roles of glycans (including glycosaminoglycans/proteoglycans) and glycan binding proteins (GBPs) (including lectins and antibodies) in biological systems have been discovered with respect to cancer, infectious diseases, and genetic disorders [7–15].

**Figure 1:**
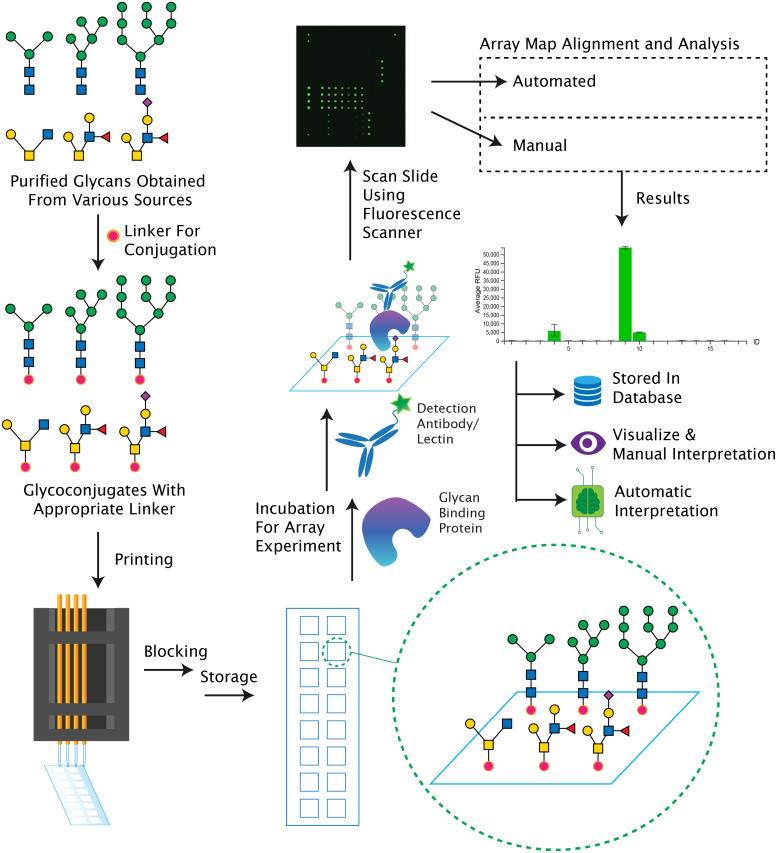
Overview of a typical glycan microarray workflow, beginning with the obtention of glycans to analysis of binding data. Briefly, glycans are chemically or enzymatically synthesized, or isolated and purified from either source materials, and then conjugated with a linker which is appropriate for the printing surface. The glycoconjugates are then printed upon appropriately functionalized slides, followed by blocking; the printed slides are stored under ideal conditions prior to experiments. Many arrays can be printed on a single slide, termed sub-arrays. The slides can then be used in a glycan microarray experiment where they are incubated with a glycan binding protein (GBP), such as lectin, antibody, or serum, virus, etc., followed by addition of a detection reagent, if the primary analyte was not fluorescently labeled, for example a fluorescent secondary antibody or streptavidin. After washing the slide to remove unbound material, the bound material is then identified and measured by scanning using a fluorescence microarray scanner. The image produced can then be analyzed using automated or manual methods to generate the array results. These results can in turn be stored in a database, or interpreted either manually or by automatic algorithms. The glycan structures in the figure were produced using GlycoGlyph [29].

As a technology to study glycan recognition by GBPs, glycan microarrays offer an invaluable tool, and permit examination of all types of lectins, along with antibodies. Glycans are recognized by many pathogens, including viruses and bacteria, and glycan microarrays are commonly now used to explore pathogen recognition of glycans [16–21]. Conversely, glycans from pathogens are also recognized by proteins in the human body and even produce an immune response [13,22–23]. An overview of a typical glycan microarray experiment is provided in Figure 1. The protocol involves the chemical covalent conjugation or noncovalent attachment of glycans (usually 20 up to ≈700 glycans) in multiple replicates and often at varying concentrations to a slide surface which is appropriately functionalized [24–25]. Such a slide can then be used to probe GBPs or pathogens using an ELISA-like sandwich assay at microscale. This enables a high-throughput screening of glycan-mediated interactions. In this review we describe how glycan microarrays are generated, how a typical glycan microarray experiment is carried out, the type of data generated, as well as the informatic tools either currently available or being developed, for the important but complex step of analyzing glycan microarray data. While parts of this review are specific for sequence defined glycan microarrays, which are the major type of glycan microarrays, there are other sophisticated approaches such as shotgun arrays and beam search array technologies for glycans from natural sources [26–28].

## Review

### Preparation of glycan microarrays

#### Decisions and steps before preparation

**Figure 2:**
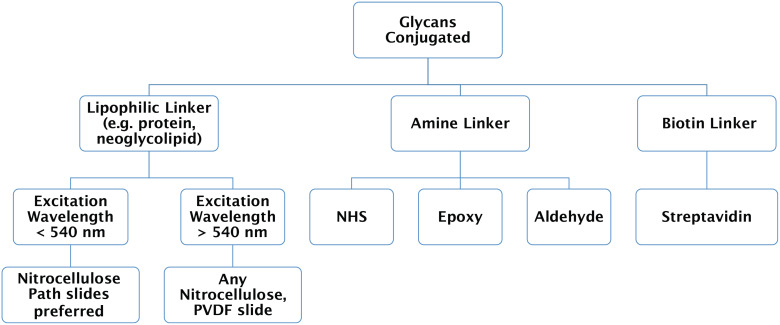
A decision tree to determine which type of surface to use depending on glycoconjugate and linker type, and scanning requirements. These linker types and surfaces are the most commonly used and there are more specialized surfaces and linker chemistries that are commercially available.

The selection of glycan microarray surface and linker is a reciprocal process, involving the preparation of the glycans in the context of appropriate surface to which the glycans are desired to be attached. Several types of functionalized slide surfaces are available such as NHS, epoxy, nitrocellulose and PVDF (Table 1); each utilizes a different mechanism of binding the ligands to the surface. Choosing an appropriate surface often depends on the type of glycoconjugates to be printed, as well as the GBP and detection wavelengths used. For example, a nitrocellulose surface has an intrinsic high background when scanned at 488 nm wavelength, thus making the surface incompatible with detection reagents which rely on this wavelength. While it is possible to use nitrocellulose slides at 488 nm wavelength with lower detector sensitivity (PMT setting) and lower scan power (laser power), it might be more advisable to check other specialized surface types (e.g., nitrocellulose PATH^®^ slides) which have lower background signals at this wavelength. A decision chart is provided in Figure 2 which can help to decide which surface would be best for a variety of situations.

**Table 1:** Summary of slide surfaces commercially available for microarrays.

Surface type	Corresponding glycoconjugate properties required	Example commercial sources

covalent conjugation		

*N*-hydroxysuccinimide (NHS)	amino functional group (primary amine preferred)	Nexterion^®^ Slide H (Schott)
epoxy	amino functional group (primary amine preferred)	Nexterion^®^ Slide E (Schott)
aldehyde	primary amino functional group	Nexterion^®^ Slide AL (Schott)

noncovalent adsorption		

streptavidin	biotin functionalization	SuperStreptavidin (Arrayit^®^)
nitrocellulose – porous type	hydrophobic, i.e., protein or lipid conjugated	ONCYTE^®^ SuperNOVA (Grace Bio-labs), UniSart^®^ 3D Nitro slides (Sartorius)
nitrocellulose – non-porous type	hydrophobic, i.e., protein or lipid conjugated	PATH^®^ (Grace Bio-labs)
PVDF	protein conjugate	SuperPVDF (Arrayit^®^)

A more detailed discussion of recent glycan linkers and surfaces was recently reported by Gao et al. [30] and McQuillan et al. [31]. Once the surface of choice is selected, the glycans need to be conjugated to an appropriate aglycone to form a glycoconjugate which can be used to link the glycan to the surface of the slide. If the glycans are already conjugated to an aglycone (for example, a glycolipid obtained directly by extraction from a natural origin), an appropriate surface needs to be selected which is compatible with the glycoconjugate.

#### Printing methods

Once the decision about the surface to which they are to be attached is made, the glycans are dissolved in appropriate printing buffers depending upon the surface chemistry involved. A microarray printer, either contact-type or non-contact-type (e.g., piezoelectric dispensing), is used to dispense small drops of 50–100 μm in diameter each onto the slide surface in a rapid manner. Usually each sample is printed as ≥4 spots/array and a single slide can have 1 or many arrays (also called sub-arrays). The concentration of the glycan in the printing buffer depends upon the efficiency of the mechanism of linking the glycan to the surface. The spots are separated from each other by a given space called the “spot pitch” (usually 2–4 times the diameter of the spots) to form distinct spots for analysis and to avoid merging of spots. Depending on the printer throughput, the number of slides/arrays to be printed and number of glycans to be printed, this process can take several hours or an entire day, and requires monitoring for accurate printing. Between the printing of each probe (glycan), there is usually a wash step which is performed to prevent any carryover for the next ligand, and this would need to be determined empirically depending on the glycoconjugates used. After the entire print run, the slides are left for incubation either in a humidified chamber at room temperature or in a cold room for several hours (or overnight), depending upon the linking mechanism. Following this incubation time, the rest of the reactive slide surface is blocked using an appropriate blocking solution and the slides are dried for storage.

### Slide storage and handling

Slides should be stored stably under appropriate conditions, depending upon the slide surface type and the linking stability, for many months. Glycan microarray slides are typically stored under vacuum sealed conditions at a cold temperature (−20 to 4 °C). When the slides are to be used, it is advised to let the slides come to room temperature without external warming in a vacuum desiccator prior to use.

### Glycan microarray experiments

If the slide is composed of several sub-arrays, the multi-well chamber method is used. If the slide is composed of 1 large array, the coverslip method can be used.

#### Multi-well chamber method

Several multi-well chambers of different array layouts are available: 8 × 3 (24-well), 8 × 2 (16-well), 8 × 1 (8-well), or 4 × 1 (4-well) chambers. The choice of the multi-well chamber to be used depends on the print layout of the arrays, which in turn would depend upon the number of glycans to be printed. The larger the number of glycans printed per array, the larger the printed area is, and hence lower number of arrays per slide. The chambers are usually made of plastic with a silicone rubber gasket that fits on top of the slide (for example, ProPlate type of multi-well chambers sold by Grace Biolabs). Such chambers allow the complete separation of a single slide into multiple wells each containing a separate sub-array that can be incubated with a sample. This enables testing of multiple samples/experiments on a single slide simultaneously with minimal sample volumes. Although usually inert to biological samples, a precautionary test should be performed to ensure that the GBP sample is compatible with the gasket/chamber materials. The chambers can be covered with parafilm or plastic plate covers to further isolate each well and prevent evaporation during assay incubation.

#### Coverslip method

When using an entire slide, the use of a multi-well chamber is impractical as it would require a large amount of GBP sample to fill the chamber. As a result an alternative coverslip method is used. In this method, a small volume of sample is placed on slide (≈70 μL), and a coverslip is placed on top to spread the sample evenly across the array surface of the slide and helps to prevent evaporation during the assay.

Detailed protocols for either method of microarray experiment and that cover different types of samples and detection methods are available on the National Center for Functional Glycomics website (https://ncfg.hms.harvard.edu/protocols), for the multi-well chamber method (e.g., NCFG slides) and for the coverslip method (e.g., CFG slides).

### Data acquisition

Once the slides are dried post sample incubation, the slides are scanned using a microarray scanner (for example, Genepix 4400A). Microarray scanners are fluorescence scanners which utilize laser technology, such that the excitation wavelength is generated by specific lasers. Commonly used laser wavelengths are 488 nm, 532 nm, 594 nm and 635 nm, which match with usual fluorophore labels on detecting reagents (e.g., labeled GBP, antibody or streptavidin). The emission wavelength of the fluorophore determines the filter used by the scanner before the intensities are measured by a photomultiplier tube (PMT) or by CCD camera. Currently, CCD camera systems are less sensitive as compared to PMT type detectors and therefore PMT systems are preferred for more accurate measurements. In addition, newer LED-based excitation systems are being developed, but are still not as sensitive and therefore laser scanners are still used. Microarray scanners scan at pixel resolutions ranging from 2.5–100 μm/pixel. This means that each pixel obtained in the final image corresponds to 2.5–100 μm on the slide depending on the resolution selected during the scan. The lower the pixel resolution value, the higher the resolution of the final image and the more data points are obtained for each spot on the slide. Thus, high-resolution images (2.5–10 μm/pixel) yield adequate data points for glycan microarray spots to provide lower standard deviations between replicate spots. The fluorescence intensity of the spots can be fine-tuned by controlling the laser power (also called LP) and the photomultiplier tube gain (also called PMT Gain). The image produced is saved as a TIFF image, usually with headers which describe the scanner settings used to acquire the image, and the intensity at each pixel is saved as the relative fluorescence units (RFU) for those scan settings.

### Spot alignment and data processing

Once the image is acquired, the image is aligned to an array map (for GenePix scanners this is called the GenePix Array List file or .gal file), which indicates the coordinates of the various spots by (row, column) numbers correlated to the material which was printed at those positions. The alignments are usually done by hand with assistance from the scanner software, which usually offers partial alignment algorithms based on background intensities. The spot diameters are also adjusted as some glycans just form smaller spots in comparison to others. Spots where information is not reliable due to extraneous factors such as poor printing due to a flaw in the surface, spot overlap/fusion with adjacent spot or presence of dust particles are flagged with a “bad” (or some numeric value) flag, so as to be disregarded in the data processing. The software then provides a results file as an output (for GenePix scanners this is the GenePix Results file or .gpr file), which contains the information of the alignment file along with spot intensity information (such as mean and median fluorescence intensity for the spot) along with information about the local background around the spot (such as mean and median background intensities). This results file is processed using Microsoft Excel in a variety of ways which usually involve consolidating data from multiple replicates of spots to provide the average background subtracted intensities in RFUs for each individual compound printed on the surface, i.e., the average of (mean spot intensity – mean background intensity) for all the replicate spots of the material for a particular excitation wavelength. This is provided along with standard deviation (SD) and coefficient of variation (SD ÷ mean) as a percentage (%CV). The results are usually presented as bar graphs with the mean or average fluorescence intensity (RFUs) on the *y*-axis with the glycan/print material id number on the *x*-axis, while the error bars represent the SD or standard error of the mean (SEM).

### Glycan microarray reporting guidelines

In order to report glycan microarray experiment and results one should follow the MIRAGE (Minimum Information Required for A Glycomics Experiment) guidelines for microarray data [32]. These guidelines cover some of the important aspects mentioned above in order for anyone to be able to reproduce the glycan microarray experiment. The current version published in June 2016 can be found at: https://www.beilstein-institut.de/download/1458/mirage_glycan_array_guidelines_version_1.0__22_june_2016.pdf.

### Tools for glycan microarray data

#### A) Repositories of glycan microarray data

**Figure 3:**
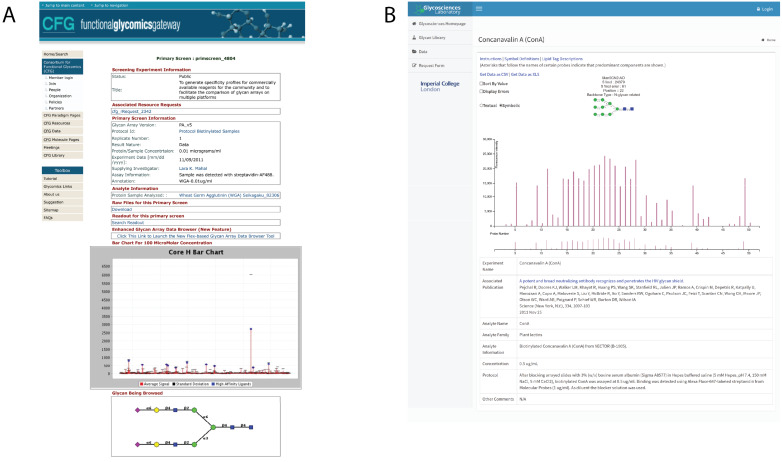
Screenshot of microarray databases: (A) Screenshot of an example of CFG glycan array data; (B) screenshot of an example of Imperial College microarray data online portal.

**1. CFG Database:** Status: Available. Address: http://www.functionalglycomics.org/glycomics/publicdata/primaryscreen.jsp. Description: The Consortium for Functional Glycomics (CFG) database of glycan array data (Figure 3A) is one of the largest archival resources for glycan microarray data. The CFG was founded in 2001 and was funded for about 10 years, during which time the main website and database were created [33]. The microarray work was then taken over by the National Center for Functional Glycomics (NCFG) which was established in 2013.

The CFG database has over 3000 experimental results files with 2 major types of arrays, the mammalian glycan printed array (noted as “Mammalian Printed Array” on the website and not to be confused with the “mammalian plate array” also present on the same page, which is not a microarray platform) and the pathogen array [34–36]. There are multiple versions of the mammalian glycan printed array (i.e., from v1 to v5.2) where each version differs by the addition or removal of some glycans from the list. The mammalian glycan arrays contain between 200–611 different glycan compounds printed per microarray. The pathogen array consists of glycans/polysaccharide isolated from bacterial sources and currently has over 300 compounds printed, with an earlier version containing fewer compounds. The database has samples consisting of animal GBPs (e.g., C-type lectins, siglecs, galectins), plant lectins, antibodies, serum samples, pathogens and microbial proteins, cells, and organisms. The website contains data available as downloadable .xlsx format along with metadata associated with the experiment, including sample and assay information.

Newer data has been challenging to add to the database, due to lack of funding support, and the use of outdated technologies which make it difficult to upgrade the current CFG database. As a result, the eventual aim is to move to a centralized microarray repository which is utilizing more modern web technologies such as that in development by GlyGen (see below) so that new data can be made easily accessible to the public.

**2. Imperial College microarray data online portal:** Status: Available. Address: https://glycosciences.med.ic.ac.uk/data.html. Description: Imperial College Microarray Data Online Portal of glycan microarray data (Figure 3B) consists of ≈160 experiments (from ≈36 publications) on a variety of microarray platforms composed of different glycans. The database includes data on antibodies, animal and plant lectins, viruses and virus-like particles, virus proteins, microbial proteins. The website is designed using newer protocols in comparison to the CFG, however, the data was classically stored in an MS Office-based platform [37] and is now being upgraded to the newer CarbArrayART database (see below).

**3. CarbArrayART:** Status: Development (available for testing upon request). Description: CarbArrayART is a software in development for the storage, processing and presentation of microarray data [38–39]. It is based on the GRITS Toolbox (classically used for mass spectrometry data) [40]. Features of CarbArrayART include storage of glycan array data from different array formats. This includes the results along with any array-specific metadata such as experiment protocol, array geometry etc. CarbArrayART will offer presentation of data with filtering and sorting functions, and generation of reports. More recently, the flexibility of the system has been improved by introducing several new input functions for sample information, experiment information and array geometries with multiple glyco-probe layouts.

**4. GlyGen microarray repository:** Status: Development. Description: GlyGen (https://www.glygen.org) is a growing resource for the inclusion of data from multiple sources for glycoinformatics [41]. A component of the project involves the creation of a glycan microarray data repository, whereby anyone can go to a website and deposit and view glycan microarray data, along with the metadata associated with the microarray experiment. Such a centralized database would greatly help the glycoscience community and help develop newer software for glycan microarray data analysis.

#### B) Data visualization/manual data analysis

**Figure 4:**
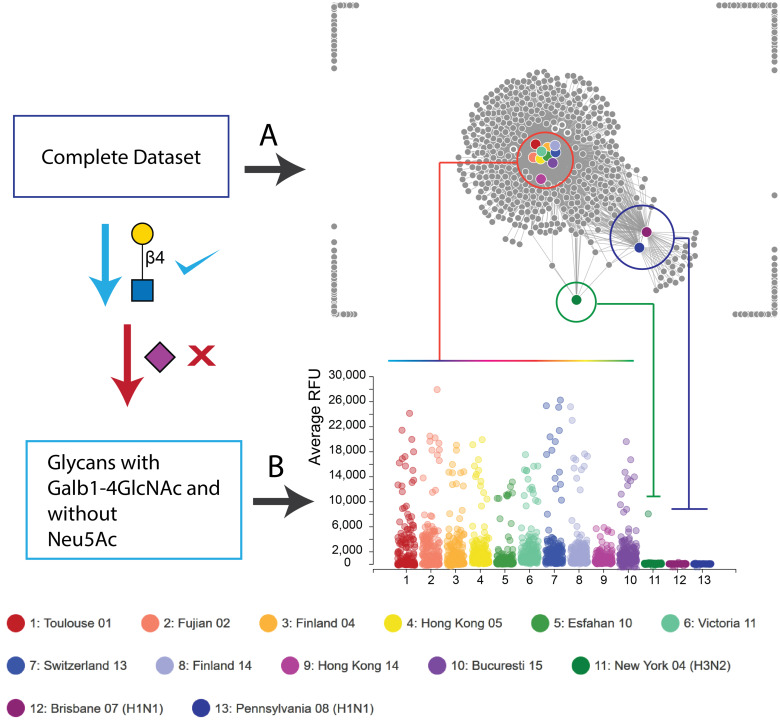
A demonstration of glycan array data visualization with GLAD. The dataset used is from Byrd-Leotis et al. [16], and is provided as a GLAD session file in the manuscript. The dataset contains data on the CFG microarray for various drift H3N2 strains of influenza (#1–10) in comparison to the distinct New York H3N2 strain (#11) and two H1N1 strains (#12 and #13). Details regarding the strains and the experiment can be found in the original paper. The legend for the colors is provided at the bottom of the figure. (A) The complete dataset can be visualized using the force graph where each strain of virus is represented by the colored circle, while each glycan is represented by a grey circle. The links between the virus to the glycan nodes are determined by the intensity of binding observed on the microarray, i.e., the higher the intensity the shorter is the link length. A short simulation is run to optimize the distances between the nodes so as to accommodate this link length and as a result of this nodes which bind similar glycans cluster together in comparison to those which bind others. Thus, the drift strains (#1–10) cluster together in the red cluster, in comparison to the New York H3N2 strain in green, all of which are separated from the purple cluster of the H1N1 strains. (B) To visualize specific binding to lactosamine (Galb1-4GalNAc) in the absence of sialic acid, the dataset is filtered for lactosamine containing glycan data and data for glycans with Neu5Ac are eliminated from the pool. A Bubble Plot of this filtered data shows clearly that the drift strains (#1–10) bind better to the lactosamine containing glycans without Neu5Ac as compared to the New York H3N2 strain and the two H1N1 strains. This analysis clearly shows the difference of the drift H3N2 strains which have acquired ability to bind lactosamine containing glycans.

**1. GLAD:** Status: Available. Address: https://glycotoolkit.com/GLAD/. Description: Traditionally, glycan array data was shared only as excel files which are non-interactive and often troublesome to visualize glycan structure alongside data visualizations such as bar charts. Yet manual data analysis is still widely used to deduce most information based on glycan microarray data. The GLycan Array Dashboard (GLAD) is a tool to visualize, analyze, compare and mine glycan array data. The tool allows users to visualize glycan array data alongside the structures using bar charts, heatmaps, calendar heatmaps, force directed graphs and correlation plots. In addition, the tool also couples some data mining features such as the ability to filter glycans by name, fragments, IDs, cutoff threshold or by rank. It also has features to sort data in particular order, normalize data and discard data points which are not present between datasets so as to get a more uniform view. All charts produced by GLAD are interactive to show the glycan structure provided the glycans are labeled using the CFG linear nomenclature system. This makes it particularly useful for manual data analysis. The plots and structures produced can directly be saved as SVG vector graphic files which can be used by most illustration software to create publication quality images [16,30–31] (Figure 4). GLAD also allows users to save and reload sessions using JSON formatted text file, which makes it easy to share the data as a GLAD session file.

#### C) Automated analysis

Automating analysis of glycan microarray data can be challenging due to the intricacies involved at multiple levels. Unlike DNA and proteins, glycans are neither linear nor template driven, making their structures computationally taxing. In addition, the complexity is compounded by the sample and experimental meta data associated with the glycan microarray experiment. The following software offer a means to address the need for automating analysis, yet it must be kept in mind that most of these software do not take into account the afore-mentioned meta data. Hence, while these software might be useful in simple use cases, even today, experts in the field prefer to use a manual methods of analysis which may be supported by some of these automated tools.

**1. GlycoPattern:** Status: Currently Unavailable – Transitioning to new host. Address: N/A. Description: GlycoPattern is a web based resource to support the analysis of glycan array data [42]. Under the hood, it utilizes the GlycanMotifMiner (GLYMMR) (https://github.com/sagravat/glymmr) algorithm [43] to perform frequent subtree mining in order to identify binding motifs. It was one of the first automated software to help mine glycan array data. GlycoPattern and GLYMMR were designed to work with CFG microarray data and hence their applicability to other datasets remains questionable. While the software was freely available earlier, at the time of this writing, the software was unavailable due to lack of funding and costs of maintaining it on university servers [44]. However, steps are being taken to try to bring the program back as an important resource to the glycoscience community.

**2. MotifFinder:** Status: Available. Address: https://haablab.vai.org/tools/. Description: MotifFinder is a graphical user interface (GUI) driven software which is able to mine glycan array data using predefined motif lists. It is a semi-automatic software in which the motifs need to be defined using MotifSpeak language which is an extension of the CFG linear nomenclature. The software is freely available for noncommercial uses, and needs to be installed in order to run. It has extensive documentation in the manual on how to perform different analysis either manually, or automated. The results output a motif table with a list of motifs, the number of glycans on the array which consist of that motif and the P-value. It also comes with other data visualizations such as box-plots, motif intensity maps, motif family membership map, list of motif glycan examples, all concentration plots, and a model structure. The software was designed to be useful for lectin and enzyme analysis. It has been used to discover fine specificities of lectins (AAL, SNA) and glycosidase enzymes (α1-2-fucosidase and an α2-3,6,8-neuraminidase) [45].

**3. SignalFinder-Microarray:** Status: Available. Address: https://haablab.vai.org/tools/. Description: SignalFinder-Microarray is an image analysis tool which allows automation to begin one step before. The software is free to use for noncommercial uses. Using this tool a user can input simply the image obtained from the scanner (.tif file) and the array map file (.gal file) and it will automatically identify and align the spots. To do this, the software uses a segment and fit thresholding algorithm, which is also useful for immunofluorescence images [46–47]. The user then has the ability to override or flag any spots to be ignored in the analysis. The software then processes the image to yield the final results either as an output which can be used with MotifFinder (see above) or as traditional average data (i.e., with mean and CV).

**4. MCAW-DB:** Status: Available. Address: https://mcawdb.glycoinfo.org. Description: MCAW-DB offers a ready-made analysis of over 1000 glycan array datasets from the CFG database (up to v5.1) via a web interface [48]. In this tool, rather than using predefined motifs, it utilizes Multiple Carbohydrate Alignment with Weights (MCAW) algorithm to align glycan structures as sequences based on their monosaccharide and linkages, and assigns each node weights depending on their binding to the ligand [49]. MCAW-DB offers a unique perspective to glycan array binding results and even takes into account gaps in structures. The tool has parameters (such as weighting) which may need to be optimized to work with other datasets, but the defaults work well with certain sample data such as lectins [48].

**5. CCARL:** Status: Available. Address: https://github.com/andrewguy/CCARL. Description: CCARL is a very new method of identifying motifs from glycan microarray experiments [50]. Previous subtree mining approaches would not account for terminal motifs. CCARL customizes the frequent subtree mining approach by extending the glycan notation to include terminal node information by including additional nodes in the graph representation to indicate the absence or presence of linkage at particular backbone carbon positions. This enables identification of terminal residues (i.e. those with all backbone carbon positions without linkages except one). In addition, it uses a new algorithm termed minimum-redundance, maximum-relevance (mRMR) to perform the subtree mining, yielding more fine-tuned results. The authors have shown the utility of CCARL on lectin data extensively. CCARL, however, does not currently have a web or GUI interface making it only possible to use it programmatically. Like other automated methods, however, the authors accept that the parameters of this method would need to be fine-tuned depending on the dataset.

#### D) Structural information tools

**1. GlyMDB:** Status: Available. Address: http://www.glycanstructure.org/glymdb/. Description: GlyMDB is a web-based database which links glycan microarray binding data from the CFG database to protein structures (PDB) [51]. A user can select a dataset from the CFG dataset available and set thresholds for binding versus nonbinding. The application can then show you motifs which make a significant binding contribution on the microarray. In addition it allows you to quickly search for PDB files with sequence identity matching to the protein sample put on the microarray along with glycan ligand length parameters. GlyMDB then retrieves the protein crystal structures for those PDB ids with protein matching the sample and glycans in the PDB structure. It allows you to view the structure in the browser to see how the glycan binds to the protein. GlyMDB thus provides a unique one-stop solution to cross referencing glycan microarray data alongside protein structure.

**2. Gly-Spec (Grafting):** Status: Available. Address: http://glycam.org/djdev/grafting/. Description: Gly-Spec (Grafting) uses structural data to predict glycan microarray binding [52]. In this software a user uploads a glycan binding protein complexed to a carbohydrate fragment in PDB format. This need not be a co-crystal structure, and can be a modeled structure as well. The application then finds glycans that contain this fragment which are present on the CFG microarray data and predicts if the protein will be able to bind them. The current limitation is that it has data only from the CFG database. Thus, building glycan array databases which are easily accessible to multiple tools can further improve the development of tools like this and help grow a field of predictive glycobiology.

## Conclusion

Glycan microarray technologies provide a wealth of information for functional glycomics, and in an efficient and decipherable manner. Once the microarrays are fabricated, the experiments can be performed within a few hours and the data analysis can be done in a day. Results from glycan microarray experiments provide needed information to develop new hypotheses about glycan recognition and function. In this article, we highlight some of the nuances of how fabrication of the glycan microarrays is done, along with tools currently available or in development to help with analysis and comparison of glycan microarray data. We identify a variety of glycan microarray repositories where interested readers can find microarray data to build new software. We also highlight manual and automated data analysis tools. One must always be aware that the intrinsic complexity of the multistep process of glycan microarray experiments means that none of the tools currently available are fool-proof and each approach and technology has strengths and weaknesses. Often, automated tools miss out on important patterns of binding which might be readily apparent to a trained individual using a manual mining approach. It is also possible that the results from automated software could be confounding rather than illuminating, if parameters of the experiment or even array fabrication (such as surface chemistry, etc.) are changed. In fact, the various chemical linkers through which glycans are attached to the array surface may dramatically change the way the glycan is presented on the microarray surface [53], thus, potentially indirectly affecting binding results. Hence, we advise experimentalists to carefully consider all of the metadata associated with each glycan array experiment. Since none of the current automation tools are flawless, the need for new tools for the analysis and reporting of glycan microarray data is ever-present.
